# Effects of Moderate Combined Resistance- and Aerobic-Exercise for 12 Weeks on Body Composition, Cardiometabolic Risk Factors, Blood Pressure, Arterial Stiffness, and Physical Functions, among Obese Older Men: A Pilot Study

**DOI:** 10.3390/ijerph17197233

**Published:** 2020-10-03

**Authors:** Wonil Park, Won-Sang Jung, Kwangseok Hong, Yae-Young Kim, Sung-Woo Kim, Hun-Young Park

**Affiliations:** 1 Department of Physical Education, Korea University, 145 Anam-ro, Seongbuk-gu, Seoul 02841, Korea; wonilpark01@korea.ac.kr; 2 Physical Activity and Performance Institute (PAPI), Konkuk University, 120 Neungdong-ro, Gwangjin-gu, Seoul 05029, Korea; jws1197@konkuk.ac.kr (W.-S.J.); kswrha@konkuk.ac.kr (S.-W.K.); 3 Department of Physical Education, Chung-Ang University, 84 Heukseok-ro, Dongjak-gu, Seoul 06974, Korea; kshong@cau.ac.kr; 4 Department of Oriental Sports Medicine, Daegu Haany University, 1 Haanydaero, Gyeongsan-si, Gyeongsangbuk-Do 38610, Korea; kyy@dhu.ac.kr; 5 Department of Sports Medicine and Science, Graduate School, Konkuk University, 120 Neungdong-ro, Gwangjin-gu, Seoul 05029, Korea

**Keywords:** combined exercise, cardiometabolic risk factors, blood pressure, arterial stiffness, aerobic performance, older population

## Abstract

We demonstrated the hypothesis that combined exercise improves body composition, cardiometabolic risk factors, blood pressure (BP), arterial stiffness, and physical functions, in obese older men. Older men (*n* = 20) were randomly assigned to combined exercise training (EXP; *n* = 10) or control groups (CON; *n* = 10). The combined exercise was comprised of elastic-band resistance training and walking/running on a treadmill and bicycle at 60–70% of maximal heart rate for 3 days/weeks. EXP showed significant decreases in body weight, body mass index, and %body fat (*p* < 0.05). The exercise program significantly reduced BP, mean arterial pressure, pulse pressure, and brachial-ankle pulse wave velocity. Furthermore, while the plasma levels of low-density lipoprotein cholesterol and epinephrine were significantly reduced in EXP, VO_2_ peak and grip strength were significantly enhanced (*p* < 0.05). In conclusion, it is indicated that 12-week regular combined exercise improves body composition, cardiometabolic risk factors, hemodynamics, and physical performance in obese older men.

## 1. Introduction

Human longevity is progressively increasing due to improvements in medical care and technology, which is a recent global phenomenon. Specifically, 8.5% of the population worldwide is over the age of 65 years, and the older population is expected to grow by 17% by 2050 [1]. Advancing age leads to functional impairments such as an inability to transfer, walk, or dress [2]. Meanwhile, it is commonly known that obesity is a major risk factor for cardiovascular diseases or metabolic disorders [3]. The prevalence of obesity in older individuals continues to rise over time. This is because older adults are prone to spending more time sitting than young people [4]. Recent evidence demonstrates that adults aged 60 years and older show significant obesity rates that exceed 37% and 39% in men and women, respectively [5].

Body composition changes with advancing age, and the redistribution of fat from peripheral and subcutaneous sources to a central location causes the waist–hip ratio to increase in older adults, with an accompanying loss of muscle mass and strength. Body mass index (BMI) is used as a surrogate for adiposity and is a useful tool in clinical practice [6]. Many studies have elucidated that overweight and/or obese individuals are related to a high incidence of cardiovascular diseases such as coronary heart disease and stroke [7]. Mechanistically, obese condition-induced endothelial dysfunction, vascular smooth muscle cell proliferation, or excessive sympathetic nerve activity result in arterial stiffness and in turn hypertension [8,9]. Interestingly, a high waist–hip ratio (WHR) and normal BMI is correlated with cardio-metabolic dysfunction (e.g., dyslipidemia, coronary disease, and hypertension) [10]. Abdominal adiposity is thought to cause central and peripheral arterial stiffness. Toto-Moukouo et al. demonstrated that pulse wave velocity (PWV) of the upper limbs was significantly higher in obese individuals, compared with non-obese peers [11].

Exercise intervention plays an important role in reducing the severe risk of diverse diseases and contributing to a healthy lifestyle in older adults [12]. Particularly, a number of studies have shown the beneficial effect of exercise on cardiovascular risks, morbidity, and mortality [13,14]. Exercise training is generally categorized into aerobic- and resistance-exercise. Resistance exercise has beneficial effects on enhancing skeletal muscle mass, strength, and power [15,16]. However, the impacts of resistance exercise training on blood pressure (BP) and arterial stiffness remain ambiguous since the exercise intervention-mediated outcomes are inconsistent with different exercise intensities, ages, and health conditions. In contrast to resistance training, aerobic exercise consistently improves cardiopulmonary fitness and vascular health by, for example, increasing aerobic capacity and lowering BP and arterial stiffness [17]. Based on these advantages of exercise, combined resistance- and aerobic-exercise interventions have compensatory or additive effects on cardiovascular and muscular function, which are greater than either operating alone, in the obese older population. Recent studies suggest that combined resistance- and aerobic-exercise has positive effects on BP; vascular properties [18]; and brachial-ankle PWV (baPWV), one of the gold-standard measurements of an index of systemic arterial stiffness in older adults [19].

Another deteriorative factor is impaired physical function that results in falls with injuries and reduced quality of life in obese older adults. The accumulation of lipids reduces muscle quality and anti-inflammatory response, which causes muscle protein degradation and subsequently muscle atrophy [20]. A structured exercise program including aerobic, strength, and balance training is reported to attenuate aging-induced muscle pathophysiological conditions. For instance, combined exercise was much better than aerobic or resistance exercise alone to improve muscle protein synthesis and myocellular quality in obese older adults [21]. Thus, lifestyle modifications that prevent lipid accumulation and frailty with advancing age are required for a better quality of life in obese older-adult individuals.

However, to our knowledge, the question of whether combined exercise interventions ameliorate various aspects of cardiovascular health in obese older men has not been fully answered. Specifically, the combined training program for older adults has provided volume-matched aerobic- and resistance-exercise. We suppose an additional volume of aerobic exercise in combined exercise might produce greater cardiovascular health than combined aerobic- and resistance-exercise equally. Therefore, we examined the impact of 12 weeks combined resistance- and aerobic-exercise on body composition, cardiometabolic risk factors, BP, arterial stiffness, and physical functions, in obese older men only.

## 2. Materials and Methods 

### 2.1. Subjects

A total of 24 sedentary and obese older men (68.8 ± 0.9 years) were entered into the study; their physical characteristics of participants are shown in Table 1. The participants with the following conditions were included in the present study: (1) those who were not taking any medication; (2) who had a BMI of >25 kg/m^2^; and (3) who participated in only low levels of activity (no exercise performed over the last 6 months). Older men with the following conditions were excluded from the study: any poorly-controlled chronic diseases, a history of acute myocardial infarction, joint replacement or fracture of the lower limb within the previous six months, and severe cognitive disturbance. The participants gave their signed consent after sufficient explanation of the experiment and the possible adverse effects. They were then randomly assigned to a control group (*n* = 10, CON) and an exercise intervention group (*n*= 10, EXP) using a computerized random number generator. Data from the remaining four subjects were discarded due to withdrawal (*n* = 4). The required sample size was estimated using an effect size (ES = 0.49) reported in the effect of exercise on cardiopulmonary fitness [22]. With an alpha of 0.05 and the desired power of 0.80, the total sample size necessary to achieve statistical significance was estimated to be 12 participants. The sample size calculation was performed using G*Power software (version 3.1.9.7, Heinrich-Heine-Universität, Düsseldorf, Germany). This study was approved by the Institutional Review Board (IRB-201812-HR-288), and all study procedures were followed were in accordance with the Helsinki Declaration.

### 2.2. Study Design

The experimental design involved the following: 1-day pre-testing, 12-week session of intervention period, and 1-day post-testing. The 12-week training session performed two days’ intervals between pre- and post-testing to avoid testing and/or training effects.

On the pre- and post-testing days, all participants underwent fasting for more than 8 hours, and after stabilization, venous blood was collected between 7:00 and 8:00 am. Then, after a 30-minute break, body composition, BP, and arterial stiffness parameters were measured in order in the morning. After lunch, the physical performance parameters (grip strength and peak oxygen uptake; VO_2_ peak) were measured in the afternoon.

The participants in EXP performed the following three kinds of combined exercise interventions for 90–120 min. Aerobic exercises were conducted after elastic-band resistance exercise in order of training program. On the other hand, CON did not perform any intervention. All exercise interventions were performed at a constant temperature and humidity (22 °C, 60%) for a total of 12 weeks, three times a week. All older obese men performed elastic-band resistance training sessions consisting of squat, incline chest press, seated row, push press, split squat, and pull apart. All participants performed 3 sets of 10–15 repetitions at an exercise intensity ranging from 6–7 on the OMNI-Resistance Exercise Scale of Perceived Exertion (OMNI-RES AM; from 0 = extremely easy to 10 = extremely hard). This range has been reported to correspond to exercise intensity levels ranging from 60%–70% of 1 RM, with a rest for 90 s per set. The elastic-band resistance training sessions were conducted for approximately 30–40 min. For aerobic exercises on a treadmill and a bicycle, the participants in EXP calculated the maximal heart rate (HRmax) using the Tanaka formula (208 – (0.7 × age)). They then performed 60 min of aerobic exercises corresponding to 60%–70% of HRmax. The order of aerobic exercise is to perform 30 min of aerobic exercise on the bicycle after 30 min of aerobic exercise on the treadmill. The participants in EXP received instructions to monitor their target heart rate by heart rate monitor (M400, Polar, Helsinki, Finland) secured on each participant’s chest during aerobic exercise session. Aerobic exercises were performed after resistance exercise with a rest for 10 min. For the 12 weeks of supervised exercise intervention, participants received specialized instruction and coaching by an experienced trainer. Therefore, participants did not have any overt events associated with the exercise intervention, but the most adverse events reported were musculoskeletal problems such as the delayed onset of muscle soreness (DOMS) and joint pain in the initial period against the exercise intervention.

### 2.3. Anthropometric Characteristics and Body Composition

Body height, body weight, body mass index (BMI), fat-free mass (FFM), and %body fat were measured using bioelectrical impedance analysis equipment (Inbody 770, Inbody, Seoul, Korea).

### 2.4. Blood Pressure

Blood pressure (BP) was measured using an automatic sphygmomanometer (HBP-9020, Omron, Osaka, Japan). All participants were examined at the same time as possible, and had a stabilization time of at least 10 min before the test. The measurement was performed twice, and the mean value was used as the measurement value.

### 2.5. Arterial Stiffness

Pulse wave velocity (PWV) is the best available parameter for assessing arterial stiffness. Brachial-ankle PWV (baPWV) was measured using an automatic oscillometric device (VP-1000plus, Omron, Osaka, Japan) after a resting period of at least 20 min. This instrument simultaneously records the baPWV and the brachial and ankle blood pressures on the left and right sides, produces an electrocardiogram, and records the heart sounds. Electrocardiogram electrodes were placed on both wrists, and cuffs were placed on the brachium and the ankles bilaterally. A microphone for detecting heart sounds was placed on the left edge of the sternum. The cuffs were connected to both a plethysmographic sensor that determined the volume pulse form and an oscillometric pressure sensor that measured blood pressure. The brachial and ankle pulse-volume waveforms were recorded using a semiconductor pressure sensor. The baPWV values on the right and left sides were obtained, and the left and right values were averaged for analysis in this study.

### 2.6. Blood Biochemistry

Venous blood variables were analyzed by the Green Cross Medical Foundation (Certified organization in The Korea Society for Laboratory Medicine). The concentrations of the following blood variables were quantified: tumor necrosis factor-alpha (TNF-α), interleukin-6 (IL-6), erythropoietin (EPO), vascular endothelial growth factor (VEGF), triglyceride (TG), total cholesterol (TC), high-density lipoprotein cholesterol (HDL-C), low-density lipoprotein cholesterol (LDL-C), free fatty acid (FFA), epinephrine, and norepinephrine. A 6- mL sample of venous blood was collected into a serum separating tube (SST) for serum. Clot formation was ensured in the SST by centrifuging the sample at 3500 rpm for 10 min. IL-6 and TNF-α were measured by an enzyme immunoassay system (Bio-Rad Lab., Hercules, CA, USA) using IL-6 and TNF-α (R & amp; D systems, Minneapolis, MN, USA) reagents based on ELISA. EPO was measured by Immulite 2000 XPI analyzer (Siemens, Eschborn, Germany) via the chemiluminescent immunoassay method. VEGF was measured by using the enzyme-linked immunosorbent assay (ELISA) method with VEGF Quantikine ELISA kits (R&D Systems, Minneapolis, MN, USA). The enzymatic colorimetric assay method was used by FFA kit (Roche, Mannheim, Germany), TG kit (Roche, Mannheim, Germany), CHOL kit (Roche, Mannheim, Germany), HDL-C plus 3rd generation kit (Roche, Germany), and LDL-C plus 2nd generation kit (Roche, Mannheim, Germany). Epinephrine and norepinephrine were measured using high-performance liquid chromatography (HPLC; Bio-Rad Lab., Hercules, CA, USA) with a plasma catecholamine kit (Roche, Mannheim, Germany). The blood assays were performed using duplicate analysis.

### 2.7. Physical Performance

The following measures of physical fitness were evaluated: grip strength was measured twice by handheld dynamometry (T.K.K 5401, Takei Instruments, Niigata, Japan) on the left and twice on the right, and each peak was recorded. VO_2_ peak was measured using the modified Bruce protocol for graded exercise testing (GXT) on a treadmill using a Vmax-229 breath-by-breath auto metabolism analyzer (SensorMedics, Yorba Linda, CA, USA).

### 2.8. Statistical Analysis

Means and standard deviations (SD) were calculated for each primary dependent variable. The normality of distribution of all outcome variables was verified using a Kolmogorov–Smirnov test. A two-way analysis (‘’group’’ × ‘’time’’) of variance with repeated measures on ‘’time’’ factor was used to analyze the effects of training programs on each dependent variable. The effect size was determined using Cohen’s d, and partial eta-squared (η^2^) values (η^2^; small, ≥0.01; medium, ≥0.06; large, ≥0.14) were calculated. If a significant interaction effect was found, a Bonferroni method post-hoc test was used to identify within-group change over time. Additionally, a paired t-test was used to compare post-intervention and pre-intervention values of dependent variables in each group separately. All analyses were performed using Statistical Package for Social Science (SPSS) version 23.0 (IBM Corp., Armonk, NY, USA), and the graphs were designed using SigmaPlot version 14.0 (Systat Software Inc., San Jose, CA, USA). A priori, the level of significance was set at 0.05.

## 3. Results

### 3.1. Body Composition

There was no significant difference in body composition between CON and EXP, before intervention. Changes in body composition in CON and EXP groups before and after intervention are shown in Table 2. The repeated two-way ANOVA analyses revealed a significant interaction (all *p* < 0.001, η^2^ > 0.437) in all of the body composition parameters. Post-hoc analyses found that while CON revealed a significant decrease (*p* = 0.013) in FFM and increase (*p* < 0.001) in %body fat, combined exercise regimen led to significant decreases in body weight (*p* = 0.001), BMI (*p* = 0.001), and %body fat (*p* < 0.001).

### 3.2. BP and Arterial Stiffness

**Figure 1:**
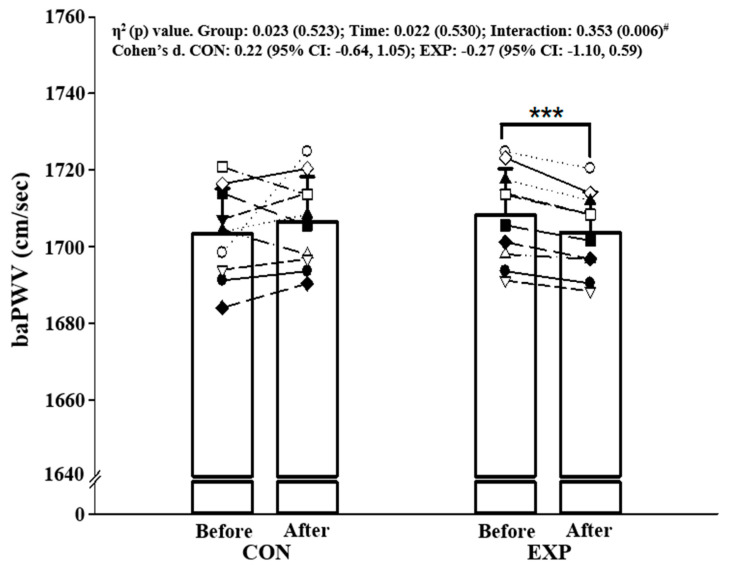
Changes in arterial stiffness before and after 12-week intervention: baPWV. Note. Values are expressed as mean (± standard deviation), CI = confidence interval, CON = control group, EXP = experimental group, each symbol (□,△,○,etc.) represents the individual participant’s pre-test and post-test values, baPWV = brachial-ankle pulse wave velocity. ^#^ Significant interaction or main effect, *** *p* < 0.001 vs. before intervention.

There was a significant difference in BP, but there were no significant differences in arterial stiffness and inflammation markers between CON and EXP before intervention. There was a significant interaction between the time and the groups (all *p* < 0.029, η^2^ > 0.239) in SBP, MAP, PP, and baPWV. In addition, it was found that a 12-week exercise intervention exclusively contributed to attenuating SBP (*p* < 0.001), MAP (*p* < 0.001), PP (*p* < 0.001), and baPWV (*p* < 0.001), in obese older participants (Table 3, Figure 1).

### 3.3. Cardionascular Risk Factors

There were no significant differences in metabolic parameters between CON and EXP, before intervention. Table 4 represents alterations in clinical blood characteristics (i.e., TG, TC, or LDL-C) and circulating factors (i.e., catecholamine) in both groups before and after the control and exercise intervention. There were significant group x time interactions (all *p* < 0.043, η^2^ > 0.208) in TG, TC, LDL-C, and epinephrine. Post-hoc analyses suggested that LDL-C (*p* = 0.008) and epinephrine (*p* = 0.026) were significantly diminished after the combined training. Conversely, the level of TG was significantly elevated (*p* < 0.019) in the absence of exercise intervention. However, inflammation markers, EPO, and VEGF did not change both groups (Table 5).

### 3.4. Cardiopulmonary Fitness and Muscular Strength

**Figure 2:**
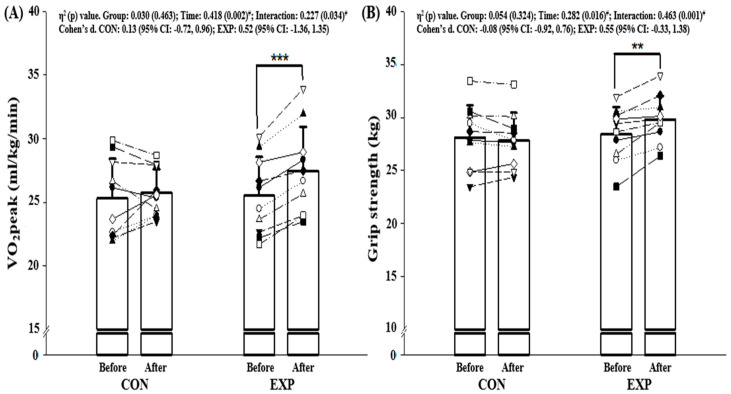
Changes in physical performance parameters before and after 12-week intervention: (**A**) VO_2_ peak and (**B**) grip strength. Note. Values are expressed as mean (± standard deviation), CI = confidence interval, CON = control group, EXP = experimental group, each symbol (□,△,○,etc.) represents the individual participant’s pre-test and post-test values, VO_2_ peak = peak oxygen uptake. ^#^ Significant interaction or main effect, ** *p* < 0.01, *** *p* < 0.001 vs. before intervention.

There were no significant differences in cardiopulmonary fitness and muscular strength between CON and EXP before the intervention. VO_2_ peak and grip strength were examined in CON and EXP groups before and after a 12-week intervention (Figure 2). According to the repeated two-way ANOVA analyses, there were significant interactions (all *p* < 0.034, η^2^ > 0.227) in all physical performance-related parameters. It was apparently shown in post-hoc analyses that the combined exercise regimen elicited substantial increases in VO_2_ peak (*p* < 0.001) and grip strength (*p* = 0.002).

## 4. Discussion

The main finding of the present study was that different volumes of moderate-intensity combined aerobic- and resistance-exercise training showed various positive effects such as body composition, cardiovascular risk factors, BP, arterial stiffness, and physical performance in older obese men.

Body weight and % body fat gradually increase, but free fat mass is reduced with advancing age [23]. These changes in body composition lead to frailty, sarcopenia, and metabolic syndrome [19]. Thus, a structural exercise program is a successful mediator to prevent the negative alterations in body composition in older adults. In this study, combined exercise training for 12 weeks significantly decreased body weight, BMI, and %body fat over the time point and increased fat-free mass. These results are consistent with previous studies showing that aerobic exercise at lactate threshold [21] and combined high-intensity exercise could reduce body weight, % body fat, and BMI for overweight and obese women [24]. While the exercise intervention group in the present study showed the tendency of increased fat-free mass after 12 weeks, the control group exhibited a considerable decrease in fat-free mass over the time point. This result was similar to previous studies in which resistance exercise alone and combined aerobic- and resistance-exercise significantly increased the fat mass more than aerobic exercise alone in overweight or obese adults [25,26].

Exercise has been suggested to improve cardiovascular health in obese individuals [27,28]. A previous study reported that BP and baPWV were reduced after combined training in older obese women [17]. In the present study, we found a reduction in SBP of 2 mmHg after 12 weeks of combined exercise intervention. Importantly, a reduction in SBP (≤5 mmHg) was demonstrated to lower risks for cardiovascular disease [29]. Our finding agrees with prior studies showing a decrease in SBP after 4 weeks of endurance training in middle-aged adults [30], whereas we observed no change in DBP after the combined exercise training. In order to profoundly address the effects of combined aerobic- and resistance-exercise on arterial stiffness in older obese adults, we felt it was necessary to measure arterial stiffness using indirect measures. A recent study has reported that the degree of obesity is significantly related to arterial stiffness [31]. We found that baPWV was significantly diminished after the combined aerobic- and resistance-exercise intervention in older obese adults. The vascular adaptation was characterized by the improved arterial stiffness with exercise intervention in older adults [17,32,33]. The results of our study were in line with previous studies suggesting that combined aerobic- and resistance-training had a beneficial effect on arterial stiffness [18]. Combined exercise improves vascular arterial stiffness in young men and women [34] and carotid arterial stiffening in young healthy men [35]. One possible mechanism underlying the attenuated arterial stiffness may be partly due to a reduction in SBP. In this context, it is implied that decreased SBP contributes to reducing arterial stiffness in obese older adults in this study. Resistance exercise training could worsen arterial stiffness in heavier subjects due to the excessive arterial wall stress [36]. However, we tried to perform aerobic exercise after resistance exercise for minimizing the stiffening. In addition, we added more time of aerobic component in combined exercise. This is consistent with the previous study, showing that aerobic training after resistance training ameliorated arterial stiffness in young adults [34]. Another mechanism is that a significant decrease in arterial stiffness following combined exercise may be attributed to increased nitric oxide (NO) bioavailability and/or decreased inflammatory responses [37]. Several studies have shown that the production of pro-inflammatory factors can be decreased by a regular exercise program. Kondo and colleges demonstrated that aerobic exercise intervention for seven months resulted in decreases in circulating levels of CRP and IL-6 in obese subjects [38]. We also found that the plasma levels of TNF- α and IL-6 were reduced after combined aerobic- and resistance-exercise, although they were statistically insignificant. This discrepancy is thought to result from the short duration of the training intervention (2-month vs. 7-month). Thus, it could be possible to propose that a combined aerobic- and resistance-exercise training regimen would help to ameliorate arterial stiffness by increasing vaso-dilatory factors in older obese individuals.

Overweight and obesity are key contributors that affect unfavorable cardiometabolic risk factors [3]. However, exercise training has beneficial effects on dyslipidemia in individuals with obesity [39]. Previous studies demonstrated that aerobic exercise interventions result in lower TG and higher HDL. We consistently found a lower TG and higher HDL-C after a 12-week combined exercise intervention. We also identified that the plasma glucose level was reduced after combined exercise intervention. Increased catecholamine is related to age-associated vasoconstriction in large conduit arteries [40]. The plasma epinephrine level was significantly decreased after training in this study. Thus, regular combined exercise can improve cardiovascular functions. Although we did not directly examine the plasma leptin that increases sympathetic nerve activity and BP, it is possible to suggest that a decrease in the leptin-catecholamine axis following combined exercise intervention may contribute to lowering BP and arterial stiffness.

The level of cardiorespiratory fitness (CRF) decreases with advancing age, but the prevalence of obesity rate is increased due to the higher exposure of inactivity [41]. Our data indicate VO_2_ peak was significantly enhanced after a 12-week intervention in the present study. This may have been caused by increased peripheral vasodilation following combined exercise training [42]. In this context, this is referred to as the “fat-but-fit paradigm”, in which individuals who are obese increase their fitness level with combined exercise training [3]. It could suggest that increased levels of CRF in older obese adults may contribute to lowering the prevalence of cardiovascular disease. An increase in muscular strength found in this study is also clinically meaningful. A weak handgrip strength and atrophy are primarily shown in obese individuals [42]. This provides evidence that muscular strength attenuates the adverse effects of overweight and/or obese on cardio-metabolic risk factors. Moreover, handgrip strength is a relevant index of cognitive decline in the older population [43]. We exhibit a significant increase in grip strength regarding the combined intervention. Older individuals with obesity are dependent on muscular strength in daily living. Taken together, combined exercise training in older obese men is an effective modality for strengthening cardiorespiratory fitness and muscular function. 

Several limitations of the present study should be considered. First, the sample size was relatively small to generalize for obese older men, so it may be best characterized as a pilot study, and we did not compare genders in the older obese population. Second, we did not compare aerobic exercise or resistance training w/wo different volumes of exercise. Third, the group was not well matched in terms of characteristics in the baseline, as there was a significant difference in blood pressure. However, the difference between the groups was fairly small. Fourth, multiple seasonal and nutritional confounding factors were not impeccably controlled throughout this study.

## 5. Conclusions

In conclusion, combined aerobic- and resistance-exercise effectively improved body composition, cardiometabolic risk factors BP, arterial stiffness, and physical functions in older obese men. These results suggest that regular participation in a combined exercise program can significantly enhance the quality of life in the older adult. Further sophisticatedly designed studies (e.g., combination of intensity, volume, as well as exercise older) are needed to determine the effect of a combined exercise intervention on health-related factors in obese older individuals.

## Figures and Tables

**Table 1:** Selected participant characteristics. Data are means (± SD).

Variables	CON	EXP	*p*-Value
Number	10	10	-
Age (years)	68.5 (0.9)	69.1 (0.9)	0.137
Height (cm)	165.8 (4.8)	164.1 (3.8)	0.409
Body weight (kg)	71.6 (5.0)	70.7 (3.8)	0.670
BMI (kg/m^2^)	26.0 (0.4)	26.2 (0.5)	0.307
Free fat mass (kg)	45.4 (3.17)	44.8 (2.4)	0.671
Body fat (%)	32.7 (1.8)	32.4 (1.4)	0.670

Note. SD = standard deviation, CON = control group, EXP = experimental group, and BMI = body mass index.

**Table 2:** Changes in body composition before and after 12-week intervention.

Variables	CON				EXP				η^2^ (*p*) Value		
	Before	After	*p*-Value	Cohen’s d (95% CI)	Before	After	*p*-Value	Cohen’s d (95% CI)	Group	Time	Interaction
Body weight (kg)	71.6 (5.0)	72.3 (5.1)	0.203	0.14 (−0.71, 0.97)	70.7 (3.8)	69.2 (4.1)	0.001 **	−0.37 (−1.20, 0.49)	0.051 (0.338)	0.096 (0.183)	0.442 (0.001) ^†^
BMI (kg/m^2^)	26.0 (0.4)	26.3 (0.8)	0.193	0.38 (−0.48, 1.21)	26.2 (0.5)	25.7 (0.6)	0.001 **	−0.93 (−1.78, −0.01)	0.037 (0.418)	0.101 (0.173)	0.451 (0.001) ^†^
Free fat mass (kg)	45.4 (3.17)	44.4 (3.1)	0.013 *	−0.31 −1.15, 0.54	44.8 (2.4)	45.2 (2.7)	0.064	0.15 (−0.70, 0.98)	0.001 (0.906)	0.117 (0.140)	0.437 (0.001) ^†^
Body fat (%)	32.7 (1.8)	34.3 (1.9)	0.000 ***	0.90 (−0.02, 1.74)	32.4 (1.4)	30.4 (1.4)	0.000 ***	−1.39 (−2.27, −0.40)	0.323 (0.009) ^†^	0.060 (0.298)	0.940 (0.000) ^†^

Values are expressed as mean (± standard deviation), CI = confidence interval, CON = control group, EXP = experimental group, BMI = body mass index. ^†^ Significant interaction or main effect, * *p* < 0.05, ** *p* < 0.01, *** *p* < 0.001 vs. before intervention.

**Table 3:** Changes in blood pressure before and after 12-week intervention.

Variables	CON				EXP				η^2^ (*p*) Value		
	Before	After	*p*-Value	Cohen’s d (95% CI)	Before	After	*p*-Value	Cohen’s d (95% CI)	Group	Time	Interaction
SBP (mmHg)	133.0 (3.4)	134.6 (3.3)	0.003 **	0.45 (−0.42, 1.28)	136.8 (2.9)	134.4 (2.5)	0.000 ***	−0.81 (−1.65, 0.09)	0.092 (0.193)	0.170 (0.071)	0.798 (0.000) ^†^
DBP (mmHg)	88.8 (4.0)	89.9 (3.2)	0.159	0.28 (−0.57, 1.12)	92.7 (3.0)	92.6 (3.3)	0.880	−0.01 (−0.85, 0.83)	0.220 (0.037) ^†^	0.097 (0.181)	0.111 (0.152)
MAP (mmHg)	103.6 (3.2)	104.8 (2.5)	0.056	0.40 (−0.46, 1.23)	107.4 (2.17)	106.6 (2.4)	0.000 ***	−0.29 (−1.12, 0.57)	0.260 (0.022) ^†^	0.027 (0.488)	0.420 (0.002) ^†^
PP (mmHg)	44.2 (4.5)	44.7 (4.2)	0.466	0.09 (−0.75, 0.93)	44.1 (4.3)	41.8 (4.8)	0.001 **	−0.56 (−1.39, 0.32)	0.034 (0.438)	0.268 (0.019)	0.429 (0.002) ^†^

Values are expressed as mean (± standard deviation), CI = confidence interval, CON = control group, EXP = experimental group, SBP = systolic blood pressure, DBP = diastolic blood pressure, MAP = mean arterial pressure, PP = pules pressure. ^†^ Significant interaction or main effect, ** *p* < 0.01, *** *p* < 0.001 vs. before intervention.

**Table 4:** Changes in metabolic parameters before and after 12-week intervention.

Variables	CON				EXP				η^2^ (*p*) Value		
	Before	After	*p*-Value	Cohen’s d (95% CI)	Before	After	*p*-Value	Cohen’s d (95% CI)	Group	Time	Interaction
TG (mg/dL)	115.7 (9.2)	119.4 (9.0)	0.019 *	0.41 (−0.46, 1.24)	116.8 (9.1)	111.1 (8.6)	0.057	−0.65 (−1.49, 0.24)	0.050 (0.344)	0.027 (0.492)	0.363 (0.005) ^†^
TC (mg/dL)	209.4 (12.2)	218.1 (11.8)	0.106	0.73 (−0.17, 1.57)	214.4 (13.9)	204.2 (12.6)	0.092	−0.77 (−1.61, 0.13)	0.056 (0.314)	0.002 (0.842)	0.273 (0.018) ^†^
HDL-C (mg/dL)	40.9 (4.2)	39.9 (1.2)	0.384	−0.28 (−1.11, 0.58)	40.8 (2.5)	42.9 (2.8)	0.092	0.77 (−0.13, 1.61)	0.104 (0.165)	0.022 (0.536)	0.176 (0.066)
LDL-C (mg/dL)	132.7 (12.5)	134.9 (7.1)	0.648	0.21 (−0.64, 1.04)	133.5 (8.2)	122.5 (7.5)	0.008 **	−1.40 (−2.27, −0.40)	0.174 (0.067)	0.126 (0.125)	0.238 (0.029) ^†^
FFA (uEq/l)	471.0 (57.4)	485.8 (43.5)	0.471	0.29 (−0.57, 1.12)	469.6 (49.2)	446.1 (40.8)	0.094	−0.51 (−1.34, 0.36)	0.067 (0.271)	0.008 (0.715)	0.130 (0.118)
Glucose (mg/dL)	114.4 (12.0)	116.5 (6.6)	0.612	0.22 (−0.63, 1.05)	120.0 (7.8)	114.3 (7.0)	0.092	−0.77 (−1.61, 0.13)	0.019 (0.559)	0.026 (0.494)	0.117 (0.140)
EP (pg/mL)	70.6 (13.1)	73.2 (10.0)	0.501	0.22 (−0.63, 1.05)	56.8 (22.9)	49.2 (19.0)	0.026 *	−0.33 (−1.16, 0.53)	0.275 (0.018) ^†^	0.059 (0.300)	0.208 (0.043) ^†^
NP (pg/mL)	267.5 (62.0)	270.8 (60.8)	0.890	0.05 (−0.79, 0.89)	240.2 (61.8)	229.2 (71.4)	0.666	−0.16 (−1.00, 0.68)	0.110 (0.154)	0.003 (0.824)	0.010 (0.679)

Values are expressed as mean (± standard deviation), CI = Confidence Interval, CON = control group, EXP = experimental group, TG = triglyceride, TC = total cholesterol, HDL-C = high-density lipoprotein cholesterol, LDL-C = low-density lipoprotein cholesterol, FFA = free fatty acid, EP = epinephrine, NP = norepinephrine. ^†^ Significant interaction or main effect, * *p* < 0.05, ** *p* < 0.01, vs. before intervention.

**Table 5:** Changes in cardiovascular risk factors before and after 12-week intervention.

Variables	CON				EXP				η^2^ (*p*) Value		
	Before	After	*p*-Value	Cohen’s d (95% CI)	Before	After	*p*-Value	Cohen’s d (95% CI)	Group	Time	Interaction
TNF-α (pg/mL)	216.2 (38.9)	234.1 (39.5)	0.228	0.46 (−0.41, 1.29)	230.9 (35.1)	214.6 (35.1)	0.163	−0.46 (−1.29, 0.41)	0.002 (0.868)	0.000 (0.929)	0.175 (0.066)
IL-6 (pg/mL)	3.6 (0.4)	3.8 (0.2)	0.206	0.66 (−0.23, 1.49)	3.8 (0.3)	3.6 (0.3)	0.009 **	−0.20 (−1.03, 0.65)	0.002 (0.847)	0.041 (0.391)	0.155 (0.086)
EPO (IU/mL)	10.9 (3.9)	11.1 (2.3)	0.891	0.07 (−0.77, 0.91)	9.5 (2.4)	11.1 (2.7)	0.068	0.63 (−0.25, 1.47)	0.029 (0.473)	0.060 (0.296)	0.036 (0.421)
VEGF (pg/mL)	33.1 (3.2)	31.6 (2.7)	0.041	−0.50 (−1.33, 0.37)	32.7 (7.1)	31.2 (3.7)	0.271	−0.14 (−0.97, 0.71)	0.003 (0.823)	0.202 (0.047) ^†^	0.000 (0.956)

Values are expressed as mean (± standard deviation), CI = Confidence Interval, CON = control group, EXP = experimental group, TNF-a = tumor necrosis factor-alpha, IL-6 = interleukin-6, EPO = erythropoietin, VEGF = vascular endothelial growth factor. ^†^ Significant interaction or main effect, ** *p* < 0.01, vs. before intervention.
